# Exploring local and global regression models to estimate the spatial variability of Zika and Chikungunya cases in Recife, Brazil

**DOI:** 10.1590/0037-8682-0027-2020

**Published:** 2020-09-25

**Authors:** Rafael Silva dos Anjos, Ranyére Silva Nóbrega, Henrique dos Santos Ferreira, António Pais de Lacerda, Nuno de Sousa-Neves

**Affiliations:** 1 Universidade Federal de Pernambuco, Departamento de Ciências Geográficas, Recife, PE, Brasil.; 2 University of Lisbon, Department of Medicine, Lisbon, Portugal.; 3 University of Évora, Department of Landscape, Environment and Planning, Évora, Portugal.

**Keywords:** Spatial Analysis, Public Health, Spatial Interaction

## Abstract

**INTRODUCTION:**

In this study, we aim to compare spatial statistic models to estimate the spatial distribution of Zika and Chikungunya infections in the city of Recife, Brazil. We also aim to establish the relationship between the diseases and the analyzed geographical conditions.

**METHODS:**

The models were defined by combining three categories: type of spatial unit, calculation of the dependent variable format, and estimation methods (Geographical Weighted Regression [GWR] and Ordinary Least Square [OLS]). We identified the most accurate model to estimate the spatial distribution of the diseases. After selecting the model that provided best results, the relationship between the geographical conditions and the incidence of the diseases was analyzed.

**RESULTS:**

It was observed that the matrix of 100 meters (as the spatial unit) showed the highest efficiency to estimate the diseases. The best results were observed in the models that utilized the kernel density estimation (as the calculation of the dependent variable). In all models, the GWR method showed the best results. By considering the OLS coefficient values, it was observed that all geographical conditions are related to the incidence of Zika and Chikungunya, while the GWR coefficient values showed where this relationship was more noticeable.

**CONCLUSIONS:**

The model that utilized the combination of the matrix of 100 meters, kernel density estimation (as the calculation of the dependent variable) and GWR method showed the highest efficiency in estimating the spatial distribution of the diseases. The coefficient values showed that all analyzed geographical conditions are related to the illnesses’ incidence.

## INTRODUCTION

Over the last decade, some viral diseases have gained notoriety due to their serious consequences for infected people in many countries, becoming a global health problem. Among the viruses causing some of these diseases are the Zika virus (ZIKV) and the chikungunya virus (CHIKV). Both viruses are transmitted primarily by *Aedes* mosquitoes^1^. 

It is important to note that a variety of ecological and economic factors could contribute to arbovirus epidemics in Brazil. One example is poor sanitation infrastructure, which is common in the country and usually leads to the creation of several mosquito breeding sites^2^. Even in poor neighborhoods, there is a high spatial variability in the cases of arbovirus diseases. Conditions related to vector proliferation and infection by arboviruses are complex and involve both individual and environmental characteristics that vary from place to place^3^. 

By identifying where the Zika and the chikungunya disease cases are located, public authorities can apply insecticides for mosquito control primarily in high‐risk areas, decreasing the transmission of these viruses. Furthermore, public educational campaigns encourage communities at risk to engage in preventive behaviors, as people who are aware of the risk in their neighborhood are more likely to eliminate potential breeding sites in their homes, apply insect repellent, dress appropriately to avoid bites, and avoid the outdoors during mosquito feeding hours^4^.

Zika fever is an exanthematous disease, related to Dengue, West Nile, and Yellow fever. This infection can last one week, with symptoms similar to Chikungunya and Dengue, including mild fever rash, arthralgia, arthritis, myalgia, headache, conjunctivitis, and edema. Severe cases involving hospitalization are uncommon, and deaths are rare^7^.

Chikungunya is another arbovirus illness caused by the chikungunya virus that belongs to the genus *Alphavirus* of the Togaviridae family. It is transmitted predominantly by *Aedes aegypti* and *Aedes albopictus mosquitoes*. However, in some areas, transmission by *Culex, Mansonia*, and *Anopheles* species has also been observed^14^. Around 50-97% of individuals infected with chikungunya develop clinical disease with fever and arthralgia^8^.

Since Zika and chikungunya have the same conditions to their proliferation, such as transmission vector, this study grouped the notified cases of these illnesses. 

Given the above, we aim to compare spatial statistic models to estimate the spatial distribution of Zika and chikungunya infections in the city of Recife (Pernambuco, Brazil). In addition, we also aim to establish the relationship between the illnesses’ incidence and the analyzed geographical conditions.

## METHODS

### Study Area

Recife, located in the northeast of Brazil, is the country’s fifth largest urban agglomeration and capital of the state of Pernambuco. Its urban layout is predominantly constituted by the coast and the urban rivers^5^ *.* Recife has 1,637,834 million residents in an area of 218.4 km² ^6^. 

### Data 

The Health Secretary of Recife provided all information on Zika and chikungunya cases, which were confirmed by diagnostic clinic and laboratory. From 2015 until 2017, 4,861 cases (124 cases of Zika and 4,737 cases of chikungunya) were geocoded. About 17% of all cases were not geocoded due to inconsistency of information related to the address. Cases notified in penitentiaries were eliminated from the geocoding.

The explanatory variables included in the analysis were based on three categories:

- *Social/Economic*: persons per household, head of household income, population density, which were provided by the Brazilian Institute of Geography and Statistics (IBGE).
- *Environmental/Infrastructural*: non-building structures, slope and green spaces. The two first factors were provided by the Light Detection and Ranging (LIDAR), with 1 m of spatial resolution. Green spaces were delimited by the Normalized Difference Vegetation Index (NDVI) from Sentinel satellite images with 10 m of spatial resolution. In all raster maps, the mean of the values was calculated for each spatial unit (census areas or matrix with cells of 100 m).
- *Climatic Factors:* Annual rainfall data from 2015 until 2017 was provided by the Brazilian Center for Monitoring and Early Warnings of Natural Disasters (CEMADEN).

The dependent and independent variables were grouped according to the spatial unit adopted in Recife. All these variables were transformed in an index, according to the following equation:

Index VI= Vi -   Va Vstd  (1)

Here, *V*_*i*_ is the value of the independent variable in the spatial unit *i, Va* is the average of the variable *V* in all census areas, and *Vstd* is the standard deviation of the variable in all spatial units.

### Spatial Units

Three main types of spatial units were utilized in this study: census areas, census areas without green spaces, and a cell matrix of 100 meters. The census area was provided by Brazilian Institute of Geography and Statistics (IBGE). For the creation of the census areas without green spaces, the green spaces in question were removed by utilizing the NDVI raster map described above. 

The cell matrix of 100 meters was created to increase the details of the values derived from the raster maps by each spatial unit. Thus, a spatial unit smaller than census areas can show more precisely spatial variability between variables (such as slope, green spaces, rainfall) and the diseases. 

### Statistical Modelling

The global regression model (Ordinary Least Squares [OLS] and the local regression model (Geographically Weighted Regression [GWR]) were applied to explore risk conditions and their relationship with the spatial distribution of Zika and chikungunya cases in Recife. The OLS is a method of least square that comes from the fact that these estimates minimize the sum of squared residuals^17^. The technique of linear regressions takes no account of location in its analysis of relationships between variables^9^ ^,^ ^10^ ^,^ ^11^. 

The GWR method is an alternative that utilizes regression, by adding the location to all variables^12^. Thus, GWR coefficients values can show where the relationship between the independent and dependent variables is more significant. The GWR function is based on the following:

$$Y_{i}=\;\sum X_{ij}\beta_{j\;}\left(p_{i}\right)+\;{ℇ\;}_{i}$$ (2)

In the presented formula, *i* is the geographical location of the *i* ^th^ spatial unit; *Y* is the dependent variable; *X* is a matrix containing a set of independent or predictor variable; ℇ is a random vector whose distribution is N (0, σ^2^ ); and β_j_ is a vector of regression coefficient of the variable *j* ^12^. Here *p*_*i*_ is the geographical location of the *i*th spatial unit. These *β*_*j*_ *(p*_*i*_ *)* would themselves contain coefficients to be estimated. 

It is important to highlight that the estimates of the coefficients of each spatial unit are related to the bandwidth adopted (quantity of spatial unit adopted or distance of the location *i*). 

The models analyzed were divided by combining three categories: spatial unit (as described above), calculation of the dependent variable, and method to estimate the diseases (GWR or OLS). 

The calculation of the dependent variable was divided in four: number of cases by each spatial unit and the mean of the kernel density by spatial unit, with radius of 500, 700 and 900 meters. 

Three parameters were used to identify the efficiency of the models: Sum of Residuals, AICC and the adjusted R^2^. 

The sum of residuals (S. Res) is the absolute sum of the squared residuals, derived by the difference between estimates and observed values.

The Akaike Information Criterion Correction (AICC) is a measure of model performance for comparing different regression models. This parameter and the sum of residuals are appropriate to identify the best model method of the regression (global or local). 

The last parameter, adjusted R^2^ is a measure of goodness of fit. Its value varies from 0.0 to 1.0, with higher values being preferable. It was used because, when an extra explanatory is added to the model, the denominator is not altered, but the numerator is. Calculations for the adjusted R^2^ value normalize the numerator and denominator by their degrees of freedom, solving the alteration problem. In this study, the adjusted R^2^ was utilized to identify the best model, while the AICC and S. Res were adopted to identify the best method of each model (GWR or OLS).

To analyze the global and local relationship between the variables and the diseases, it was necessary to choose the best model according to the adjusted R^2^. Thus, it was possible to identify the intensity and spatial variability - utilizing the GWR method - of the coefficient values for each variable.

Three statistical variables were used to analyze the relationship between the variables and the diseases in the OLS method: robust probability,Variance Inflation Factor (VIF), and the Koenker Statistic. The robust probability indicates whether a coefficient is statistically significant (p < 0.01). The VIF values (>7.5) indicate redundancy among explanatory variables. If the Koenker Statistic indicates values smaller than 0.01, the relationships modeled are not consistent (either due to non-stationarity or heteroskedasticity)^10^.

## RESULTS

By analyzing the results in Table 1, it was observed that the cell matrix of 100 meters showed the best efficiency to estimate the diseases in both methods (OLS and GWR), excepting Model 3, which utilized the number of cases by cell matrix of 100 meters. By comparing the adjusted R^2^ between Model 1 (census areas as spatial unit) and Model 6 (cell matrix of 100 meters), it is possible to notice an improvement of 0.09 to 0.35 (OLS method) and 0.47 to 0.92 (GWR method).

**TABLE 1:** Models categorized by combination of spatial unit, calculation of dependent variable, and method to estimate the Zika and chikungunya cases.

Model	Method	Bandwidth (m)	Spatial Unit	Dependent Variable	Adjusted R2	S. Res	AICC
1	OLS		census areas	number of cases	0.092	1 134	5 024
	GWR	500	census areas	number of cases	0.47	593	37
							
2	OLS		census areas without green spaces	number of cases	0.095	1 133	5 022
	GWR	500	census areas without green spaces	number of cases	0.47	595	47
							
3	OLS		cell matrix of 100 meters	number of cases	0.11	9 121	52 935
	GWR	500	cell matrix of 100 meters	number of cases	0.19	7 280	14 110
							
4	OLS		cell matrix of 100 meters	kernel density estimation (500 m)	0.36	9 461	45 912
	GWR	500	cell matrix of 100 meters	kernel density estimation (500 m)	0.89	2 631	5 135
							
5	OLS		cell matrix of 100 meters	kernel density estimation (700 m)	0.367	9 717	45 767
	GWR	700	cell matrix of 100 meters	kernel density estimation (700 m)	0.92	2 743	7 067
							
6	OLS		cell matrix of 100 meters	kernel density estimation (900 m)	0.359	9 949	45 886
	GWR	900	cell matrix of 100 meters	kernel density estimation (900 m)	0.92	2 855	8 036

In respect to the calculation of the dependent variable, the results which presented higher adjusted R^2^ values numbers were observed in the models that utilized the kernel density estimation. This fact highlights the difference between models that utilize number of cases by spatial unit and the mean of kernel density estimation by spatial unit. However, considering the adjusted R^2^ in OLS method, the radius of kernel density of 700 meters showed the best efficiency. Both Models 5 and 6 (with radii of 700 and 900 meters, respectively) had the same adjusted R^2^ of 0.92. 

In all models, the GWR method showed the best results, when the combination of all three parameters was analyzed: adjusted R^2^, sum of residuals, and the AICC. One particularity was that in Model 3, the OLS and GWR methods had similar adjusted R^2^ values. 

By analyzing the adjusted R^2^ values of the models, in general, it was identified that the best results were shown in Model 5. This model had the spatial unit of cell matrix with 100 meters and kernel density with a radius of 700 meters. 

The importance of the coefficients and parameters presented in the OLS method lies in the possibility of identifying how many variables were spatially significant and which variables were more significant to estimate the diseases. However, it is worth noting that these parameters show the global relationship between the geographical conditions and the diseases. 

In this case, in the OLS method, it was observed that the VIF was low, indicating no problems with multicollinearity in all variables. Thus, this parameter shows that no variables were redundant. 

Another point to consider is that the robust probability showed significant spatial autocorrelation for all variables. This significant spatial autocorrelation shows that the all variables improved the estimates of Zika and chikungunya cases. 

The Koenker Statistic value for all variables was statistically significant (p <0.01), showing that relationships modeled are not consistent due to non-stationarity. This fact can indicate that the GWR model could show better estimations, leading to the thought that regression methods based on location (GWR method) are better than global regression methods to estimate the diseases, such as OLS.

In Model 5, three factors presented positive coefficients in the OLS method (Table 2): persons per households, population density, and slope. Other independent variables, such as green spaces, head of household income, nonbuilding structures and precipitation, had negative coefficients. 

**TABLE 2:** Independent variables utilized and their relationship with Zika and chikungunya cases according to coefficient, robust probability, and VIF in the OLS method (Model 5).

Variable	Coefficient	Robust Probability	VIF
Persons per household	0.320608	0.000000	1.252564
Head of household Income	-0.179351	0.000000	1.154345
Rainfall	-0.120486	0.000000	1.141473
Nonbuilding Structures	-0.120486	0.000005	1.578071
Slope	0.058705	0.000000	1.240517
Population Density	0.468012	0.000000	1.567344
Green Spaces	-0.230611	0.000000	2.547128

**FIGURE 1:**
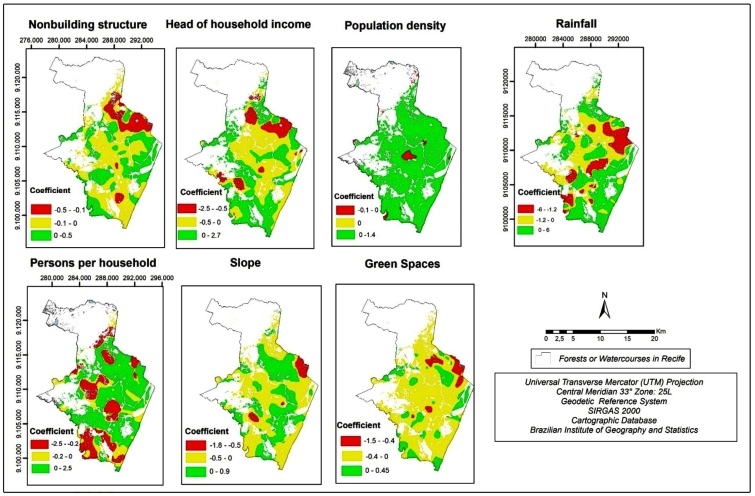
Maps of GWR coefficient values divided by the geographical conditions analyzed.

By considering the coefficients’ spatial distribution of GWR method, it is possible to identify which variables presented spatial heterogeneity. Thus, it enables the recognition of disparities between GWR and OLS coefficient values. By analyzing the GWR coefficients in Figure 1, it is possible to identify that their values can be different according to the geographic location. Although the independent variables showed similarities between GWR and OLS coefficients, some regions presented an inverse relationship. 

Although the coefficient values of the OLS method presented an inverse relationship between non-building structures and the diseases, it is possible to see that some regions (east and southwest, for example) presented positive coefficient values in GWR method. However, negative coefficient values were found in most spatial units (62%). The coefficient values in GWR varied from -0.5 to 0.5, while the coefficient value in the OLS method was -0.12.

The negative coefficients for the variable head of household income in the OLS model was confirmed by the GWR coefficient values in most census areas. By comparison, the coefficient value in OLS method was -0.17, while the coefficient values in GWR method varied from -2.5 to 2.7. However, about 54% of the spatial units showed negative coefficient values in GWR method. 

By analyzing population density, in most spatial units, the coefficient value in GWR model is positive, mainly where the disease rates were high, confirming the coefficient values in the OLS model (0.46). At the center of the municipality, one of the regions showed lower population density but higher Zika and chikungunya rates than its neighboring areas. This fact resulted in a negative coefficient value in the GWR map. The coefficient values in GWR method varied from -0.1 to 1.4. Despite that, about 96% of the spatial units showed positive coefficient values. 

Regarding the relationship between arbovirus diseases and persons per household, in most spatial units, the coefficient was positive in the GWR model (57%), agreeing with the coefficient values in the OLS model. It is important to note that, although some spatial units presented high persons per household rates, the arbovirus rates were low, causing a negative coefficient in GWR model. 

The relationship between slope and arboviruses presented positive and negative coefficients, without a spatial distribution pattern. However, about 60% of spatial units showed negative coefficients. This result differs from the OLS coefficient values. 

The inversely proportional relationship between green spaces and arboviruses found in the OLS method is also present in all spatial units. The areas where the highest positive coefficient values occurred (mainly in the northeast of the city) showed low presence of green spaces and low Zika and chikungunya rates. About 81% of spatial units showed negative coefficient values in the GWR method. 

Regarding the rainfall variable, the OLS coefficient values showed that regions with lower precipitation rates had higher Zika and chikungunya rates. However, the coefficient values in the GWR method presented negative values for 50% of the spatial units studied. 

**FIGURE 2:**
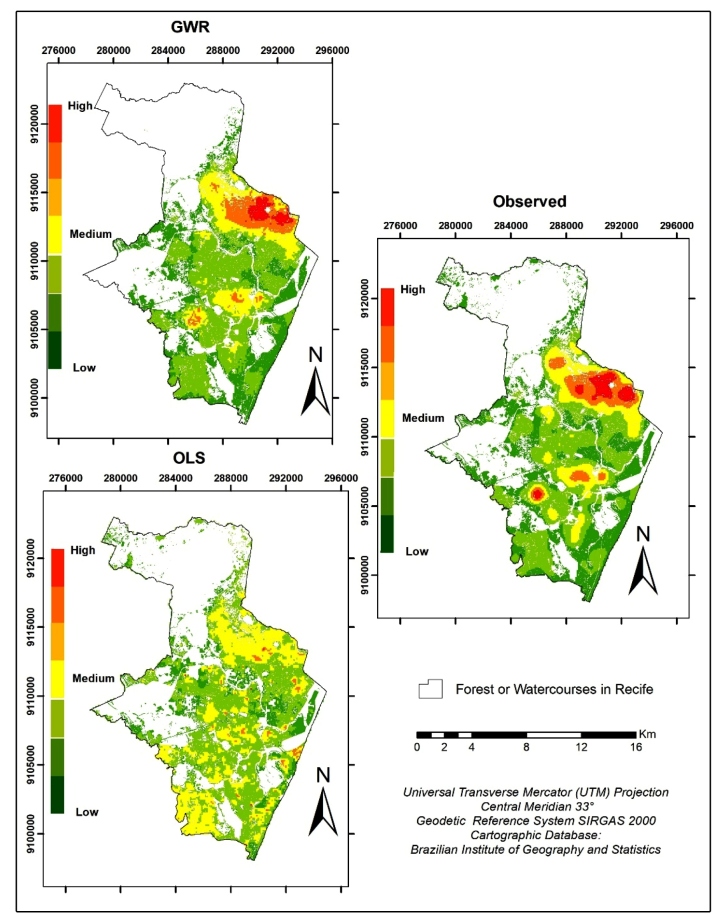
Density of estimated (GWR model and OLS model) and observed cases of Zika and chikungunya in Recife, Brazil.

By analyzing the differences between the estimated and observed cases of Zika and chikungunya in Figure 2, some particularities became evident. The OLS method presented the greatest difference when compared to the observed data, to which the GWR method showed similarities in the spatial distribution of the arboviruses. However, it was possible to identify the spatial variability of the diseases in both models, where the highest Zika and chikungunya rates were observed in the northeastern and some central areas of Recife.

## DISCUSSION

By analyzing the results in Table 1, it was observed that the efficiency of the models can be assessed according to the spatial unit, the calculation of dependent variable, and the method of the model. 

By analyzing the different parameters utilized in all models, it was possible to identify some particularities. The adoption of spatial units smaller than census areas can improve the estimates of the diseases studied. Some geographical conditions, which derive from the raster maps, have high spatial variability inside of the census areas. Thus, by adopting the cell matrix of 100 meters as the spatial unit instead of the census areas, the relationship between these conditions and the diseases can be more precise, improving the adjusted R^2^ values. 

Another point to consider is that census areas without green spaces as the spatial unit does not improve the calculation of the population density, resulting in best estimates. 

The calculation of the dependent variable was another important parameter to estimate the Zika and chikungunya rates. The models that adopted the mean of kernel density to calculate the Zika and chikungunya rates presented better estimates than models that utilized the number of cases by spatial unit. This fact shows that the methods utilized (OLS and GWR) have a sensitivity related to the spatial variability of the diseases. By adopting the number of cases, their spatial variability was abrupt between the spatial units, decreasing the proximity with the spatial variability of the independent variables. This occurs because the variations of the geographical conditions are smooth, demanding this behavior of the dependent variable. It is important to note that the adoption of the mean of kernel density can be justified by two factors: (a) the best efficiency to estimate the Zika and chikungunya cases, utilizing the adjusted R^2^ values as parameter; and (b) the regionalization of the rates.

Regarding the search radius adopted (500, 700 and 900 meters) in kernel density, the adjusted R^2^ values were similar in all models. However, the model that utilized the search radius of 700 meters presented slightly better efficiency than others models. 

The third parameter analyzed in the model were the OLS and GWR methods. In all models, the GWR method showed the best results, according to the analysis of the sum of residuals, AICC, and adjusted R^2^ values. Although both methods estimate a dependent variable, their purposes are different. When the results of the OLS method estimates are good, the coefficient values are an important aspect to analyze the global relationship between the geographical conditions and the diseases. However, the Koenker Statistic value in Model 5 indicated that the coefficient values can be very different according to location, justifying the GWR method. On the other hand, the GWR method can indicate where the relationship was more notable or inverse of the OLS coefficient values. 

Others studies showed that this efficiency in GWR method is better than the OLS method. For example, the correlation between some factors and notifications of Dengue in Pakistan was 0.37 in OLS model and 0.49 in GWR model^13^. An aspect to consider is that these correlations can vary in space and time. For example, the correlation between population density, slope, altimetry, and malaria varied from 0.47 to 0.83, between 2001 and 2007^14^. 

By considering all the parameters discussed and their efficiencies to estimate the Zika and chikungunya cases, in general, Model 5 presented the best results to estimate the diseases. This model had a cell matrix of 100 meters as spatial unit and mean of kernel density with 700 meters of search radius as calculation of the dependent variable. 

By exploring the coefficients of both the OLS and GWR methods, some particularities were noteworthy. 

Regarding to the GWR and OLS coefficient of nonbuilding structures, the negative coefficient values can be explained because in areas where the distance among buildings is low, the opportunities of the mosquito to infect the households are increased. Another point to consider is that slum areas have the lowest rates of nonbuilding structures in Recife, justifying the correlation between the highest rates of Zika and lowest rates of nonbuilding structures. It is important to note that the building density can be an indicator of the temperature due to the latent heat that is absorbed in areas with high building density. 

A relationship between income and spatial distribution of Zika and chikungunya cases was observed in both coefficients methods (OLS and GWR). However, income is not a predominant factor in some census areas in the north of the city. This fact could be related to the decrease of arboviruses cases in areas where the low-income population is located. The heterogeneity of variables related to arboviral disease is observed in other studies, in which some socioeconomic indicators were not statistically significant in categorizing risk areas^3^. However, studies showed that the income rate is the factor that best characterized the risk areas for arbovirus diseases such as Dengue, for example^15^. 

The relationship between population density and some diseases can be explained by other studies that revealed areas with higher human population densities had lower socioeconomic position, poorer water and sanitation conditions, and frequent tire capping facilities, but had reasonable infrastructure^16^. For example, in Porto Rico, it was identified that the poor population, population density, urbanized areas, and high temperature presented a correlation of 0.78 with incidence of Zika, in GWR model^17^. Another study in Pakistan verified that among variables such as altimetry, distance from rivers and population density, the last factor showed the highest correlation with notifications of Dengue^13^.

In general, the correlation between persons per household and incidence of Zika and chikungunya diseases in both methods showed a directly proportional correlation. Results were detected in Ecuador, where the relation between Zika and persons per households was statistically significant^18^. In Venezuela, it was observed that the relation between Dengue and persons per household was directly proportional^19^. 

Historically, the regions with high slope degree or flat areas (such as mangrove) are inhabited by a low-income population in Recife^5^. This fact resulted in a high spatial variation of the GWR coefficient values. In any case, the OLS method showed positive coefficients, highlighting that the regions with high slope degree are more susceptible to proliferation of Zika and chikungunya cases in Recife.

Another point to consider is that the mosquito *Aedes aegypti* prefers urbanized areas where the presence of vegetation cover is more limited. Thus, the green spaces can be a factor correlated with urbanization and, consequently, related to spatial distribution of the arboviruses. This result is similar to the study that identified the highest chikungunya incidence is in regions with highest percentages of urbanized land in Rio de Janeiro^2^. 

A second factor to highlight is that the *Aedes aegypti* preferences for human blood decrease with the increase of vegetation cover. Moreover, it is important to highlight that most slum areas are highly compact, displaying the highest roof coverage and low vegetation cover rate^20^. 

Some models identified that, while an increase in temperature should shift the *Aedes aegypti* boundaries towards higher latitudes, the increase of rainfall would be detrimental to its enlargement^21^. In most studies that analyzed the relationship between climatic parameters and arboviruses, it was identified that these studies are related to the arboviruses temporal distribution, disregarding the spatial distribution, due to the lack of meteorological well-distributed stations across the cities. However, the temporal analyses indicate that extremely intense rainstorms that produce a high volume of precipitation in a few hours may flush out larvae, leading to decreased vector abundance and arbovirus transmission^2^. These observations can suggest that the regions with low precipitation volume are better for the proliferation of mosquitoes. 

Given the above considerations, the model that utilized the combination of the matrix of 100 meters (as the spatial unit), kernel density estimation (KDE) (as the calculation of the dependent variable) and Geographical Weighted Regression (as the estimation method) showed the highest efficiency to estimate the spatial distribution of Zika and chikungunya cases. 

It is important to note that, although the GWR method presented best results, regression linear methods, such as OLS, can show the global relationship between geographical conditions and some diseases. On the other hand, the GWR coefficient values show where this relationship is more evident. 

By comparing the GWR and OLS coefficient values in Recife, three geographical conditions showed a notable agreement in most spatial units in both methods: non-building structures, population density, and green spaces (more than 60% of spatial units). However, in the OLS method, it was possible to identify that all geographical conditions were relevant to the model.
